# Ultra-low-field MRI for bedside imaging of severe multiple sclerosis

**DOI:** 10.1007/s00415-026-13907-w

**Published:** 2026-06-03

**Authors:** Niels Bergsland, Alex Burnham, Michael G. Dwyer, Alex Bartnik, Ferdinand Schweser, Cheryl Kennedy, Ashley Tranquille, Mehak Semy, Ella Schnee, Suyog Pol, Aaron Krawczyk, David Young-Hong, Svetlana Eckert, David Hojnacki, Christine Reilly, Ralph H. B. Benedict, Bianca Weinstock-Guttman, Robert Zivadinov

**Affiliations:** 1 Buffalo Neuroimaging Analysis Center, Department of Neurology, Jacobs School of Medicine and Biomedical Sciences, University at Buffalo, State University of New York, 77 Goodell Street, Suite 450, Buffalo, NY 14203 USA; 2 The Boston Home, Dorchester, MA USA; 3 Center for Biomedical Imaging, University at Buffalo, State University of New York, Buffalo, NY USA; 4 Department of Neurology, Jacobs Comprehensive MS Treatment and Research Center, Jacobs School of Medicine and Biomedical Sciences, University at Buffalo, State University of New York, Buffalo, NY USA

**Keywords:** Multiple sclerosis, Severe MS, Ultra-low-field MRI, High-field MRI, Gray matter, Thalamus, Cortical, Atrophy, Disability

## Abstract

**Background:**

Severe multiple sclerosis (MS) presents challenges for clinical research due to patient mobility constraints and specialized care needs. Traditional MRI studies often exclude this population but portable, ultra-low-field (ULF) MRI at 0.064 T enables bedside imaging.

**Objectives:**

To (i) compare ULF MRI volumetry with standard 3 T, (ii) assess the capability of portable ULF MRI in characterizing severe MS.

**Methods:**

This prospective study enrolled two cohorts. Cohort 1 included healthy controls (HC) and people with MS (pwMS). Cohort 2 enrolled individuals with progressive MS, with either severe or less severe MS. Both cohorts underwent ULF MRI while those in Cohort 1 were also scanned on 3 T MRI. Clinical assessments included disability and cognition. MRI data was processed using both conventional (e.g., SIENAX) and AI-driven (e.g., WMH-SynthSeg) techniques. Group comparisons and MRI-clinical associations were assessed.

**Results:**

Cohort 1 included 14 HCs (39 ± 13 years, 11 females) and 33 pwMS (32 ± 10 years, 24 females). Cohort 2 included 24 severe MS individuals (62 ± 10, 15 females) and 16 less severe MS individuals (61 years ± 11, 8 females). MRI passed quality control except for scans from 2 individuals in Cohort 2. In Cohort 1, 3 T and ULF MRI showed robust disease differences, with strongest effect sizes for ULF MRI-derived whole brain (partial η^2^** = **0.264), cortical gray matter (GM) (partial η^2^** = **0.234), and thalamic volumes (partial η^2^** = **0.266) from WMH-SynthSeg. In terms of severe versus less severe disease in Cohort 2, the largest effect sizes were obtained with SIENAX-derived cortical GM volume (partial η^2^** = **0.349) and whole brain volume (partial η^2^** = **0.290), while WMH-SynthSeg yielded the highest effect size for WM volume (partial η^2^** = **0.209). For clinical outcomes, associations were dependent on processing method, although SIENAX yielded the most consistent correlations in Cohort 2.

**Conclusion:**

Portable, ULF MRI is feasible in pwMS and provides insights into late-stage neurodegeneration in severe MS.

**Supplementary Information:**

The online version contains supplementary material available at 10.1007/s00415-026-13907-w.

## Introduction

A subset of people with multiple sclerosis (pwMS) experience a particularly severe form of the disease, which may be evident early after onset or develop later, following two disability trajectories [1–4]. In early aggressive MS, individuals reach an Expanded Disability Status Scale (EDSS) score of ≥ 6.0 within 10 years from disease onset or before age 40 [5], whereas those with late severe MS attain similar disability levels after more than 10 years of disease duration or after 40 years of age [2–4]. Early aggressive MS typically results in rapid and marked disability, with an estimated prevalence of 5–10% among pwMS [1, 5]. Late severe MS progresses more gradually, likely being influenced by aging-related processes [2–4, 6, 7]. These individuals eventually develop an even more advanced state of disability, marked by severe ambulatory impairment and/or substantial cognitive decline and often require wheelchair or bed confinement, assistance with daily activities, psychosocial support, rehabilitation, and professional care [2].

Little research has been conducted to characterize severe MS, in part because there are many practical difficulties in studying this population [2–4]. Those living with severe MS are often confined to their own homes or skilled nursing facilities, where constant, intensive care is available [2–4]. The high-dependency nature of their conditions means that transporting them to academic research facilities for participation in studies is not only a logistical challenge but also a potential health risk. The need for specialized transport and caregivers to accompany them may lead to prohibitive costs and overall infeasibility. In fact, many pwMS with a severe form of the illness do not obtain MRI or other examinations for monitoring disease activity once they progress to this stage. Furthermore, clinical and academic study settings are generally unequipped to manage the complex needs of these individuals, who may require medical equipment, emergency care, or additional accommodations, increasing the difficulty of conducting longitudinal studies.

In the Comprehensive Analysis of Severely Affected Multiple Sclerosis (CASA-MS) phase 1 study, we showed significantly lower cortical gray matter (GM) and thalamic volume in severe compared to less severe MS (defined as those not meeting the severe criteria outlined earlier) [4]. Moreover, no significant differences between the two groups were observed in terms of lesion volume, suggesting that GM atrophy may be more relevant for the development of severe disability. A major weakness of that retrospective study was that MRI data were collected from different local MRI facilities for those with a severe phenotype, leading to uncontrolled heterogeneity in MRI outcomes while the less severe group was acquired on the same 3 T scanner. This was inevitable as ambulatory and cognitive disabilities rendered it logistically infeasible to transport them to specialized MRI centers.

Portable, ultra-low-field (ULF) MRI technology presents a promising opportunity for studying the vulnerable population of individuals with severe MS, as it offers a means to safely and effectively gather imaging data right at their bedside, minimizing distress and maximizing comfort [8, 9]. ULF MRI (e.g., 64 millitesla [0.064 T]) systems have been cleared by the Food and Drug Administration and offer greater accessibility and cost efficiency compared to traditional scanners [8, 10, 11]. However, ULF images are also of comparatively lower quality, with reduced signal-to-noise ratios and spatial resolution compared to standard MRI scanners. To overcome this limitation, artificial intelligence (AI) techniques have been developed to improve image quality and facilitate volumetric segmentation from ULF MRI acquisitions [10, 12–15].

To address the limitation of heterogeneous MRI acquisitions in the CASA-MS Phase 1 study, we assessed the feasibility of using standardized, ULF MRI to image fully disabled individuals with the goal of analyzing tissue volumetry data obtained from a recently proposed AI segmentation technique as well as a conventional method. The primary aim was to determine whether ULF-derived tissue volumetry could capture previously reported group differences between severe and less severe MS, and to compare results with respect to standard 3 T MRI in an independent cohort. For the latter aim, we hypothesized that the ULF AI-derived volumes would show stronger effects compared to those from a conventional algorithm. Based on our preliminary findings, we hypothesized that individuals with severe MS would exhibit lower GM volume compared to those with less severe disease.

## Materials and methods

The study was approved by the University at Buffalo (UB) Institutional Board and informed consent was obtained from all participants.

### Cohort 1: ULF MRI and 3 T MRI study population

From an ongoing study in our institute, 47 consecutive subjects, 33 pwMS and 14 HC, group-matched for age and sex, were selected for comparison of ULF and 3 T MRI. The inclusion criteria were 1) age between 30 and 65 years, 2) MS diagnosis with the 2017-revised McDonald criteria [16], and relapsing–remitting MS [17]. Exclusion criteria were: 1) relapse or steroid use within 30 days prior to study enrollment or MRI, 2) pregnant or lactating individuals, 3) inability to obtain informed consent.

All participants were imaged on both ULF 0.064 T MRI (Hyperfine Swoop) and 3 T systems (MRI Philips MR7700) within a max of 28 days.

### Cohort 2: CASA-MS phase 2 ULF MRI Study population

The CASA-MS phase 2 was a prospective, cross-sectional study conducted at The Boston Home (TBH), a specialized residential MS facility in Dorchester, Massachusetts, and at a tertiary MS center at the UB, Buffalo, New York. The cohort enrolled 24 severely disabled pwMS from TBH. The study inclusion criteria were: 1) age between 30 and 80 years, 2) MS diagnosis with the 2017-revised McDonald criteria [16], 3) progressive MS [17], and 4) early aggressive disability accrual of severe MS (disease duration < 10 years or age < 40 years when reaching EDSS ≥ 6.0) or late severe disability accrual of MS (disease duration ≥ 10 years or age ≥ 40 years when reaching EDSS ≥ 6.0). Sixteen age-, sex-, and disease duration matched progressive pwMS from UB with less severe disease were prospectively enrolled (separate from Cohort 1) in the same period. Exclusion criteria were: 1) relapse or steroid use within 30 days prior to study enrollment or MRI, 2) pregnant or lactating individuals, 3) inability to obtain informed consent from study participant or medical proxy.

All participants in Cohort 2 were imaged on the same ULF 0.064 T MRI system.

### Clinical assessments

A standardized questionnaire was used to collect demographic and clinical information with oversight from a board-certified neurologist (BWG).

In both cohorts, pwMS were assessed using EDSS [18], Timed 25-foot walk test (T25FWT) [19], and Nine-hole peg test (9HPT) [20]. In Cohort 2, the Scripps Neurological Rating Scale (SNRS) was utilized [21], along with the Combinatorial Weight-Adjusted Disability Score (CombiWISE) [22]. A maximum of 180 and 300 s were allowed to complete the T25FWT and 9HPT, respectively. Lower SNRS and higher Combi-WISE scores indicate greater disability.

Participants in both cohorts underwent neuropsychological testing assessments under the supervision of a board-certified neuropsychologist (RHBB). Cognitive function was assessed via Symbol Digit Modalities Test (SDMT) [23] and the Auditory Test of Processing Speed (ATOPS) [24].

### 3 T MRI acquisition

For Cohort 1, all pwMS were scanned on the same 3 T system (MRI Philips MR7700) with sagittal 3D T1-weighted (w) (repetition time (TR): 9.6 ms; echo time (TE): 4.5 ms, 0.73 × 0.73 × 0.75 mm^3^ voxels) and T2w-fluid-attenuated inversion recovery (FLAIR) (TR: 8000 ms, TE: 300 ms, inversion time (TI): 2400 ms, 0.74 × 0.74 × 0.56 mm^3^ voxels) acquisitions (without gadolinium).

### ULF MRI acquisition

**Fig. 1:**
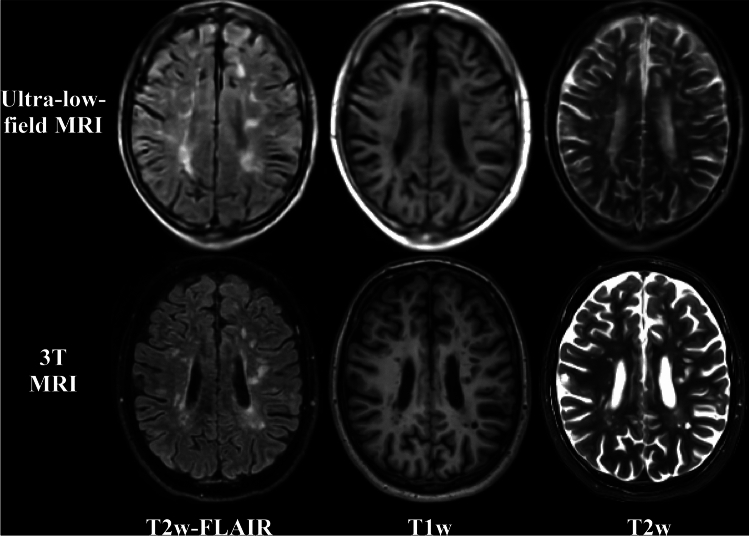
Ultra-low-field (ULF) and 3 T MRI in a 62-year-old male with late severe MS (EDSS 7.5). Figure shows native FLAIR, T2 and T1-weighted sequences on ULF (top row) and 3 T (bottom row) MRI. Due to different slice thicknesses and geometric distortions, perfect anatomical matching between the ULF and 3 T is infeasible

In both cohorts, all pwMS were scanned on the same ultra-low-field 0.064 T MRI scanner (Hyperfine Swoop, Guilford, CT, USA). The imaging protocol matched MS studies [15, 25], with axial and sagittal T1w, axial T2w, and axial and sagittal T2w-FLAIR images (Fig. 1). The resolutions were 1.5 × 1.5 × 5 mm^3^ (T1w and T2w) and 1.6 × 1.6 × 5 mm^3^ (T2w-FLAIR). Scanning parameters can be found elsewhere [15, 25]. Only axial acquisitions were utilized for processing.

**Fig. 2:**
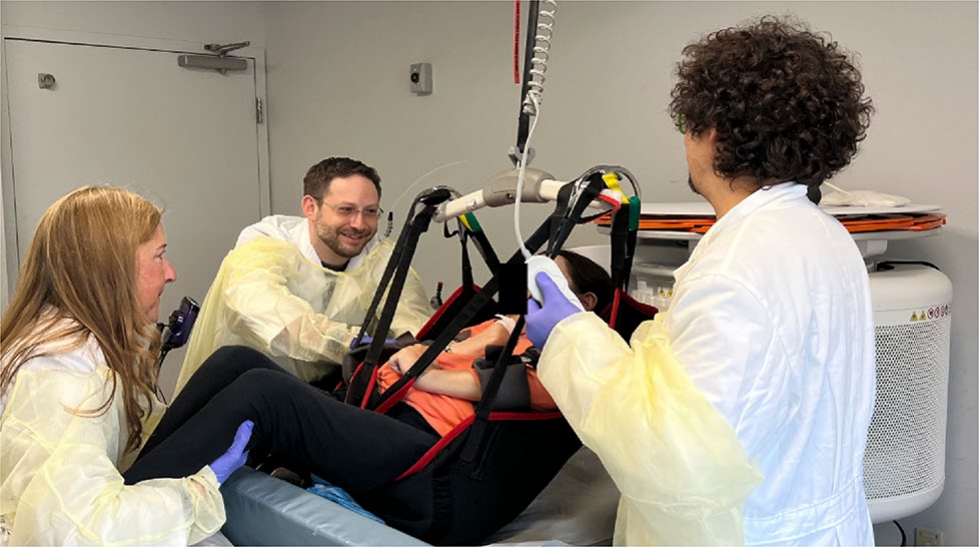
An FDA-cleared Hyperfine Swoop MRI ultra-low-field MRI (64mT), allowing for imaging of people with severe multiple sclerosis at the bedside, is shown. The Hoyer lift, which was used to safely move the individual from his wheelchair to the MRI table and facilitate proper positioning into the head coil

To scan severely disabled pwMS in Cohort 2 at TBH, we used a Hoyer lift to move participants onto the MRI table (Fig. 2).

### MRI processing

Analysis was performed in a fully blinded manner by a single reader (NB, 24 years of experience). For Cohort 1, participant data from ULF and 3 T MRI was analyzed independently and in a random order.

#### Processing common to both scanners

T2 lesion volume (LV) and T1-LV were obtained using an in-house deep learning-based classifier trained without data from either the 3 T Philips or ULF Hyperfine scanner and then corrected manually using a contouring/thresholding technique [26]. Tissue volumes were determined with lesion-filled [27]. T1w images using SIENAX (https://fsl.fmrib.ox.ac.uk/fsl/docs/#/structural/siena/index) [28]. For ULF data, we replaced BET with SynthStrip (https://surfer.nmr.mgh.harvard.edu/docs/synthstrip/) [29] but kept BET for skull mask generation. We obtained measures of whole brain (WB), cortical GM, white matter (WM), and ventricular volumes as well as a volumetric scaling factor to control for head size.

#### Processing exclusive to data from the 3 T scanner

Lesion-filled T1w images were processed using FreeSurfer (version 7.4.1, https://surfer.nmr.mgh.harvard.edu), yielding measures of WB, cortical GM, WM, ventricular volumes, as well as total intracranial volume (TIV).

#### Processing exclusive to data from the ULF scanner

T1w were processed using WMH-SynthSeg from FreeSurfer (version 7.4.1, https://surfer.nmr.mgh.harvard.edu/fswiki/WMH-SynthSeg) [30], which is designed for processing ULF scans of low resolution. WMH-SynthSeg does not enhance raw image quality; it is a post-processing and segmentation method that yields strong correlations with 3 T volumetry. Inpainting was not used as WMH-SynthSeg explicitly handles the presence of WM lesions. We obtained the same volumes as in the SIENAX/FreeSurfer processing while also measuring thalamic volume.

#### Data availability

The data that support the findings of this study are available upon reasonable request to the corresponding author.

### Statistical analyses

All statistical analyses and visualizations were performed using SPSS version 28.0 (IBM, Armonk, NY, USA) and RStudio version 2023.09.1 + 494 (R Foundation for Statistical Computing, Vienna, Austria). ATOPS underwent log transformation due to non-normality of the data; all other variables were found to have a normal distribution.

Reliability between manually corrected lesion volumes obtained using ULF and 3 T MRI was assessed using a two-way mixed-effects intraclass correlation coefficient model for absolute agreement based on single measurements [ICC(A,1)]. Because of the difficulty in accurately co-registering ULF and 3 T MRI due to several reasons (e.g., large differences in nonlinear geometric distortions and slice thickness), Dice scores were not calculated, in line with other studies that have compared ULF segmentations with those obtained from higher field MRI [30].

For group comparisons, we utilized analysis of covariance (ANCOVA), adjusting for age, sex, and either head size or TIV. All assumptions required for ANCOVA were assessed and confirmed to be adequately met prior to conducting the analyses. We used Pearson correlation between volumes obtained from 3 T and ULF MRI and between post-processing methods. Partial correlations, adjusted as in ANCOVA, assessed relationships between clinical (EDSS, SNRS, Combi-WISE, SDMT and ATOPS) and MRI outcomes. Multiple comparisons were addressed using the Benjamini–Hochberg false discovery rate (FDR) procedure. FDR correction was applied separately within prespecified families of tests, defined as all variables tested within the primary group comparison, all variables tested within each subgroup comparison, and all imaging measures tested in association with each clinical outcome. Results with FDR-corrected p-values < 0.05 were considered statistically significant.

## Results

### Demographic and clinical characteristics of the study populations

Table 1 shows demographic and clinical characteristics of study participants from both Cohorts. HCs and pwMS in Cohort 1 were largely similar in all assessments except for significantly worse SDMT (*p* = 0.018) and T25WT (*p* = 0.008) performance in the latter. In Cohort 2, participants with severe MS had increased disability, as evidenced on all clinical assessments (all p < 0.001). The prevalence of cardiovascular comorbidities was similar between the two groups, except for a trend for higher prevalence of hypertension in those with less severe MS (*p* = 0.053). Demographic and clinical characteristics between early and late disability accrual were similar, except for numerically worse clinical outcomes in the early group for SNRS, Combi-WISE and SDMT (all *p* < 0.088).

**Table 1:** Demographic and clinical characteristics of the cohorts

	*Cohort 1*			*Cohort 2*					
Demographic and clinical characteristics	HC (*n* = 14)	MS (*n* = 33)	*p* value	Less severe MS (*n* = 16)	Severe MS (*n* = 24)	*p* value	Early disability accrual severe MS (*n* = 12)	Late disability accrual severe MS (*n* = 12)	*p* value
Age in years, mean (SD)	38.7 (13.2)	32.3 (10.0)	0.308	60.6 (11.0)	62.0 (9.5)	0.673	62.2 (11.7)	61.8 (7.3)	0.918
Age at onset in years, mean (SD)	–	27.2 (7.6)	N/A	32.4 (10.9)	31.7 (10.3)	0.830	34.3 (13.3)	29.1 (5.5)	0.226
BMI, mean (SD)	28.9 (6.7)	26.6 (5.9)	0.260	26.7 (4.0)	26.2 (4.7)	0.712	26.6 (5.1)	25.8 (4.4)	0.672
Education, years, mean (SD)	16.7 (2.4)	14.6 (2.4)	**0.011**	14.5 (2.7)	14.7 (2.9)	0.724	14.7 (3.6)	14.6 (2.6)	0.474
Disease duration (years), mean (SD)	–	13.8 (8.4)	N/A	30.1 (10.7)	30.3 (8.4)	0.962	27.9 (9.4)	32.7 (6.9)	0.087
Female, n (%)	11 (78.6)	24 (72.7)	1.00	8 (50.0)	15 (62.5)	0.522	9 (75)	6 (50)	0.400
Race, n (%)			0.326			0.999			0.999
White	10 (71.4)	23 (69.7)		15 (93.8)	23 (95.8)		12 (100)	11 (91.7)	
African American	2 (14.3)	9 (27 .3)		1 (6.2)	1 (4.2)		0 (0)	1 (8.3)	
Asian	1 (7.1)	0 (0.0)		0 (0)	0 (0)		0 (0)	0 (0)	
Other	1 (7.1)	1(3.0)		0 (0)	0 (0)		0 (0)	0 (0)	
Marital status, *n* (%)	UN	UN	N/A			** < 0.001**			0.172
Single				2 (12.5)	8 (33.3)		2 (16.7)	6 (50)	
Married				12 (75)	4 (16.7)		2 (25)	1 (8.3)	
Divorced/separated				0 (0)	10 (41.7)		5 (41.7)	5 (41.7)	
Widowed				2 (12.5)	2 (8.3)		2 (16.7)	0 (0)	
Disease course, n (%)	–		–			0.228			0.187
Relapsing-progressive		28 (84.8)		9 (56.3)	7 (29.2)		5 (41.7)	2 (16.7)	
Non-relapsing progressive		5 (15.2)		6 (37.5)	15 (62.5)		7 (58.3)	8 (66.7)	
Primary-progressive		0 (0.0)		1 (6.3)	2 (8.3)		0 (0.0)	2 (16.7)	
Comorbidities, *n* (%)									
Hypertension	1 (7.1)	5 (15.2)	0.452	10 (62.5)	7 (29.2)	0.053	4 (33.3)	3 (25)	0.999
Hypercholesterolemia	2 (14.3)	4 (12.1)	0.839	10 (62.5)	10 (41.7)	0.148	4 (33.3)	6 (50)	0.680
Heart disease	0 (0.0)	1 (3.0)	0.510	7 (43.8)	4 (16.7)	0.08	1 (8.3)	3 (25)	0.590
Diabetes	0 (0.0)	0 (0.0)	N/A	2 (12.5)	7 (29.2)	0.395	4 (33.3)	3 (25)	0.640
Obesity	5 (35.7)	7 (21.2)	0.297	3 (18.8)	6 (25)	0.717	4 (33.3)	2 (16.7)	0.999
Smoking	2 (14.3)	6 (18.1)	0.745	7 (43.8)	9 (37.5)	0.947	4 (33.3)	5 (41.7)	0.999
Relapses in the last 24 months, mean (SD)	N/A	0.2 (0.5)	N/A	0.2 (0.5)	0.05 (0.2)	0.332	0.09 (0.3)	0 (0.0)	0.205
EDSS, median (IQR)	N/A	2.5 (1.5–3.5)	N/A	4.0 (3.1–6.0)	8.0 (8.0–8.5)	** < 0.001**	8.3 (8–8.5)	8.0 (7.5–8.5)	0.121
9HPT in s, mean (SD)	18.8 (2.4)	24.6 (10.8)	0.081	25.3 (4.7)	223.5 (106.6)	** < 0.001**	210.3 (113.6)	236.8 (102.4)	0.554
T25FWT in s, mean (SD)	4.6 (0.7)	5.9 (2.2)	**0.008**	18.6 (43.2)	180 (0.0)	** < 0.001**	180 (0.0)	180 (0.0)	N/A
SNRS, mean (SD)	UN	UN	N/A	71.8 (16.1)	36.1 (11.5)	** < 0.001**	32.8 (10.5)	39.3 (12.1)	0.086
CombiWISE, mean (SD)	UN	UN	N/A	33.6 (12.7)	78 (6.5)	** < 0.001**	79.9 (5.8)	76.2 (6.8)	0.088
SDMT, mean (SD)	63.4 (6.7)	54.8 (16.0)	**0.018**	49.6 (12.3)	23.5 (10.4)	** < 0.001**	19.1 (10.6)	27.4 (9.0)	0.083
ATOPS, mean (SD)	1.46 (0.06)	1.38 (0.45)	0.681^	1.51 (0.045)	1.74 (0.15)	** < 0.001**	1.77 (0.17)	1.72 (0.14)	0.468
DMT efficacy, *n* (%)	N/A		N/A			** < 0.001**			0.217
No therapy		5 (15.1)		3 (18.8)	21 (87.5)		9 (75.0)	12 (100.0)	
Moderate		2 (6.1)		8 (50.0)	3 (12.5)		3 (25.0)	0 (0.0)	
High		26 (78.8)		5 (31.3)	0 (0.0)		0 (0.0)	0 (0.0)	

*p* values were derived from the chi-square test, Student’s t-test, and Mann–Whitney U test. In bold are shown significant *p* values < 0.05

^ ATOPS was assessed in 6 healthy controls and 16 individuals with multiple sclerosis, respectively

*MS* multiple sclerosis, *SD* standard deviation, *BMI* body mass index, *EDSS* Expanded Disability Status Scale, *IQR* interquartile range, *9HPT* Nine-Hole Peg Test, *T25WT* timed 25-foot walk test, *UN* unavailable, *N/A* not applicable, *SNRS* Scripts Neurological Rating Scale, *Combi-WISE* Combinatorial Weight-Adjusted Disability Score, *SDMT* Symbol Digit Modalities Test, *ATOPS* auditory test of processing speed, *DMT* disease-modifying treatment

### Feasibility of ULF MRI

In Cohort 1, all study participants were successfully imaged (i.e., WB coverage and minimal motion artifact) on both the 3 T and ULF systems. In Cohort 2, two individuals with severe MS were unable to be properly positioned within the head coil due to MS-related postural spasticity of the neck and/or shoulders, resulting in incomplete coverage.

### Reliability of manually corrected lesion segmentations

ICCs were excellent for both T2-LV (0.92, confidence interval: [0.87–0.96], *p* < 0.001) and T1-LV (0.87, confidence interval: [0.78–0.93], *p* < 0.001).

### MRI differences in Cohort 1

Table 2 shows MRI outcomes between HC and pwMS in terms of volumetric outcomes obtained using different post-processing algorithms and different MRI systems. After correction for multiple comparisons, all measures were significantly different between the groups except for ventricular volume as assessed using SIENAX on the ULF T1w image. In terms of LVs, the greatest effect sizes were seen with the fully automated WMH-SynthSeg ULF T2w-FLAIR (partial η^2^ = 0.239) and WMH-SynthSeg ULF T1w (partial η^2^ = 0.127) for T2-LV and T1-LV, respectively. Effect sizes for the semi-automated methods were similar regardless of the imaging system utilized. Unlike with the fully automated ULF T1-LV assessment, no T1 hypointensities were found in the HC group with either 3 T or ULF data. With respect to tissue volumes, the largest effect sizes for WB (partial η^2^ = 0.264), thalamic volumes (partial η^2^ = 0.266), and cortical GM volumes (partial η^2^ = 0.234) were found with WMH-SynthSeg run on ULF T1w images while those for WM (partial η^2^ = 0.180) and ventricular volumes (partial η^2^ = 0.131) were found with 3 T SIENAX and FreeSurfer, respectively. In terms of SIENAX-derived data, effect sizes were largely similar between 3 T and ULF assessments. Correlations between tissue volumes obtained from different segmentation techniques and field strengths are shown in the Supplement Table.

**Table 2:** Differences in MRI outcomes between healthy controls and people with multiple sclerosis in Cohort 1

MRI-based measures	HC (*n* = 14)	MS (*n* = 33)	Partial η^2^ [95% CI]	*p* value
T2-lesion volume				
3 T Semi-automated	0.4 (0.4)	11.8 (15.8)	0.137 [0.002–0.315]	**0.018**
ULF Semi-automated	0.2 (0.3)	10.5 (12.7)	0.157 [0.013–0.354]	**0.015**
ULF WMH-SS (FL)	5.7 (1.9)	12.1 (5.9)	0.239 [0.052–0.436]	**0.005**
T1-lesion volume				
3 T Semi-automated	0.0 (0.0)	3.8 (6.2)	0.100 [0.000–0.290]	**0.039**
ULF Semi-automated	0.0 (0.0)	4.8 (6.7)	0.126 [0.004–0.321]	**0.021**
ULF WMH-SS (T1)	3.0 (0.8)	8.7 (8.2)	0.127 [0.004–0.322]	**0.021**
Whole brain volume				
3 T SIENAX	1128.6 (107.2)	1062.0 (110.5)	0.239 [0.051–0.438]	**0.005**
3 T FreeSurfer	1102.6 (108.0)	1008.3 (113.4)	0.189 [0.025–0.390]	**0.009**
ULF SIENAX	1082.6 (84.8)	1006.9 (117.3)	0.196 [0.028–0.397]	**0.008**
ULF WMH-SS (T1)	1051.0 (105.2)	951.8 (102.7)	0.264 [0.067–0.460]	**0.005**
Cortical gray matter volume				
3 T SIENAX	482.5 (55.1)	458.8 (48.8)	0.094 [0.000–0.284]	**0.045**
3 T FreeSurfer	463.2 (58.2)	430.1 (47.8)	0.112 [0.000–0.310]	**0.031**
ULF SIENAX	401.1 (35.1)	367.6 (50.2)	0.154 [0.011–0.354]	**0.015**
ULF WMH-SS (T1)	457.4 (49.4)	418.5 (47.8)	0.15 [0.013–0.358]	**0.015**
Thalamic volume				
3 T FIRST	14.8 (1.5)	12.7 (2.2)	0.209 [0.033–0.412]	**0.007**
3 T FreeSurfer	15.2 (1.9)	12.7 (2.3)	0.199 [0.30–0.400]	**0.008**
ULF WMH-SS (T1)	14.3 (2.0)	11.7 (2.0)	0.220 [0.040–0.420]	**0.007**
White matter volume				
3 T SIENAX	506.7 (53.8)	469.4 (58.0)	0.18 [0.021–0.381]	**0.010**
3 T FreeSurfer	473.1 (47.0)	426.6 (60.5)	0.144 [0.008–0.343]	**0.017**
ULF SIENAX	550.1 (46.8)	514.7 (57.0)	0.178 [0.020–0.379]	**0.010**
ULF WMH-SS (T1)	431.7 (44.0)	393.1 (51.7)	0.262 [0.065–0.459]	**0.005**
Ventricular volume				
3 T SIENAX	25.4 (8.1)	36.6 (24.3)	0.100 [0.000–0.292]	**0.040**
3 T FreeSurfer	17.5 (6.5)	27.6 (26.0)	0.131 [0.004–0.329]	**0.021**
ULF SIENAX	31.6 (9.4)	43.7 (24.6)	0.079 [0.000–0.264]	0.065
ULF WMH-SS (T1)	19.9 (6.6)	27.0 (22.0)	0.152 [0.011–0.351]	**0.015**

All measures are reported as mean (standard deviation) with absolute volumes reported in milliliters. *p* values and effect sizes (Partial η^2^) were derived from analysis of covariance models correcting for age and sex. For tissue volume comparisons, either head size for SIENAX measures or intracranial volume for WMH-SynthSeg measures was included as an additional covariate. In bold are shown significant *p* values < 0.05 that survived false discovery rate (FDR) correction with the Benjamini–Hochberg procedure

*CI* confidence interval, *ULF WMH-SS* WMH-SynthSeg, *T1* T1-weighted, *FL* T2-weighted fluid-attenuated inversion recovery

### MRI differences in Cohort 2

Table 3 shows MRI outcomes in pwMS in severe versus less severe disease and early versus delayed accrual of disability using different post-processing algorithms with ULF data.

**Table 3:** Differences in MRI outcomes between people with multiple sclerosis in Cohort 2

MRI-based measures	Less severe MS (*n* = 16)	Severe MS (*n* = 22)^%^	Partial η^2^ [95% CI]	*p* value	Early disability accrual severe MS (*n* = 10)^%^	Late disability accrual severe MS (*n* = 12)	Partial η^2^ [95% CI]	*p* value
T2-lesion volume								
Semi-automated	23.8 (20.9)	27.5 (15.9)	0.013 [0.000–0.167]	0.605	22.5 (13.1)	31.6 (17.3)	0.083 [0.000–0.371]	0.526
ULF WMH-SS (FL)	17.3 (8.3)	18.8 (7.3)	0.014 [0.000–0.170]	0.605	16.5 (7.5)	20.8 (6.8)	0.099 [0.000–0.391]	0.526
T1-lesion volume								
Semi-automated	12.2 (13.8)	12.3 (8.4)	< 0.001 [0.000–0.000]	0.952	10.2 (7.5)	14.1 (9.0)	0.075 [0.000–0.361]	0.526
ULF WMH-SS (T1)	14.6 (12.3)	16.0 (8.7)	0.006 [0.000–0.0141]	0.712	14.4 (8.0)	17.3 (9.3)	0.041 [0.000–0.311]	0.634
Whole brain volume								
ULF SIENAX	978.5 (104.2)	847.4 (83.2)	0.292 [0.065–0.506]	**0.005**	801.1 (86.0)	886.0 (60.0)	0.190 [0.000–0.490]	0.404
ULF WMH-SS (T1)	855.8 (100.3)	775.1 (82.7)	0.218 [0.025–0.441]	**0.015**	734.6 (66.5)	808.9 (81.9)	0.016 [0.000–0.264]	0.781
Cortical gray matter volume								
ULF SIENAX	361.5 (40.6)	307.5 (28.0)	0.349 [0.105–0.552]	**0.002**	296.6 (29.4)	316.6 (24.4)	0.099 [0.000–0.399]	0.526
ULF WMH-SS (T1)	404.0 (50.8)	380.4 (36.7)	0.025 [0.000–0.206]	0.496	364.1 (31.9)	393.9 (36.0)	0.009 [0.000 – 0.237]	0.821
Thalamic volume^#^								
ULF WMH-SS (T1)	11.0 (1.9)	9.5 (1.4)	0.184 [0.012–0.408]	**0.027**	8.8 (1.2)	9.7 (1.5)	< 0.001 [0.000–0.000]	0.971
White matter volume								
ULF SIENAX	498.8 (56.0)	447.6 (49.3)	0.137 [0.000–0.360]	0.053	416.7 (41.5)	473.3 (40.5)	0.251 [0.000–0.541]	0.374
ULF WMH-SS (T1)	330.2 (41.3)	293.5 (40.2)	0.165 [0.006–0.390]	**0.034**	275.5 (30.5)	308.6 (42.2)	0.003 [0.000–0.188]	0.895
Ventricular volume								
ULF SIENAX	55.1 (21.1)	68.7 (24.4)	0.253 [0.042–0.473]	**0.009**	61.4 (24.9)	74.8 (23.2)	0.050 [0.000–0.034]	0.634
ULF WMH-SS (T1)	51.7 (22.8)	64.2 (28.8)	0.120 [0.000–0.340]	0.068	53.6 (25.5)	73.1 (29.5)	0.025 [0.000–0.287]	0.747

*MS* multiple sclerosis, *CI* confidence interval, *ULF* ultra-low-field, *WMH-SS* WMH-SynthSeg, *T1* T1-weighted, *FL* T2-weighted fluid-attenuated inversion recover

%Two individuals with early disability accrual in the severe MS group were excluded due to incomplete coverage of the brain

#Of the ten “Early disability accrual severe pwMS”, three failed thalamic segmentation while two failed in the “Late disability accrual severe pwMS” group

All measures are reported as mean (standard deviation) unless otherwise noted. Absolute volumes are reported in milliliters. P-values and effect sizes (Partial η^2^) were derived from analysis of covariance models correcting for age and sex. For tissue volume comparisons, either head size for SIENAX measures or intracranial volume for WMH-SynthSeg measures was included as an additional covariate. In bold are shown significant *p* values < 0.05 that survived false discovery rate (FDR) correction with the Benjamini–Hochberg procedure. FDR correction was applied separately for the “Less severe MS versus severe MS” and “Early disability accrual severe MS” versus “Late disability accrual severe MS” comparisons

We did not find significant differences in T2-LV and T1-LV between groups, regardless of the approach utilized. In terms of comparing those with severe versus less severe disease, the largest effect sizes were found with SIENAX for cortical GM (partial η^2^** = **0.349), WB (partial η^2^** = **0.292), and ventricular volumes (partial η^2^** = **0.200) (all *p* ≤ 0.023), while those for WM (partial η^2^** = **0.165) and thalamic volume (partial η^2^** = **0.192) were seen with WMH-SynthSeg (both p = 0.035). After correction for multiple comparisons, no significant differences were seen when comparing the early disability *vs.* late disability accrual groups, regardless of which method was used, although a large effect size was seen for WM volume with SIENAX (partial η^2^** = **0.251).

### Relationship between clinical outcomes and MRI measures in Cohort 1 (3 T and ULF dataset)

Table 4 shows partial correlation analyses between clinical outcomes and MRI measures in Cohort 1. After correction for multiple comparisons, T2- and T1-LV showed consistent EDSS associations across techniques and field strengths except for 3 T semi-automated T1-LV (*r* = 0.398, *p* = 0.064). The only significant association with ULF-derived tissue volumes was for WB volume obtained using WMH-SynthSeg (*r* =  − 0.488, *p* = 0.029). In terms of SDMT, the strongest associations were generally seen with ULF-derived measures apart from thalamic volume (3 T FIRST r = 0.598, p = 0.005 versus ULF WMH-SynthSeg *r* = 0.525, *p* = 0.047).

**Table 4:** Correlations between clinical outcomes and MRI measures in people with multiple sclerosis in Cohort 1

MRI-based measures	EDSS Correlation [95% CI] *p* value	SDMT Correlation [95% CI] *p* value
T2-lesion volume		
3 T Semi-automated	**0.442** **[0.104–0.689]** **0.40**	− 0.349 [− 0.686–0.110] 0.164
ULF Semi-automated	**0.525** **[0.210–0.741]** **0.012**	− 0.429 [− 0.732–0.017] 0.093
ULF WMH-SS (FL)	**0.473** **[0.142–0.709]** **0.030**	− 0.275 [− 0.640–0.191] 0.241
T1-lesion volume		
3 T Semi-automated	0.396 [0.049–0.658] 0.057	− 0.308 [− 0.660–0.156] 0.194
ULF Semi-automated	**0.453** **[0.117–0.695]** **0.038**	− 0.365 [− 0.696–0.092] 0.149
ULF WMH-SS (T1)	**0.575** **[0.277–0.772]** **0.012**	− 0.312 [− 0.663–0.151] 0.194
Whole brain volume		
3 T SIENAX	− 0.329 [− 0.616–0.036] 0.100	0.506 [0.068–0.781] 0.061
3 T FreeSurfer	− 0.339 [− 0.623–0.024] 0.098	0.521 [0.087–0.789] 0.056
ULF SIENAX	− 0.406 [− 0.668 – − 0.054] 0.057	**0.583** **[0.175–0.820]** **0.47**
ULF WMH-SS (T1)	− 0.416 [− 0.675 – − 0.065] 0.057	0.583 [0.175–0.820] 0.047
Cortical gray matter volume		
3 T SIENAX	− 0.275 [0.578–0.095] 0.147	0.342 [− 0.132–0.689] 0.180
3 T FreeSurfer	− 0.278 [− 0.580–0.091] 0.147	0.441 [− 0.016–0.746] 0.093
ULF SIENAX	− 0.414 [− 0.674 – − 0.064] 0.057	0.518 [0.083–0.787] 0.058
ULF WMH-SS (T1)	− 0.278 [− 0.580–0.092] 0.147	0.539 [0.112–0.798] 0.058
Thalamic volume		
3 T FIRST	** − 0.557** **[− 0.767– − 0.239]** **0.012**	**0.662** **[0.298–0.858]** **0.029**
3 T FreeSurfer	− 0.395 [− 0.661 – − 0.041] 0.059	0.480 [0.036–0.767] 0.078
ULF WMH-SS (T1)	** − 0.544** **[− 0.756 – − 0.229]** **0.012**	**0.556** **[0.288–0.855]** **0.029**
White matter volume		
3 T SIENAX	− 0.285 [− 0.585–0.084] 0.147	0.461 [0.009–0.757] 0.084
3 T FreeSurfer	− 0.332 [− 0.618–0.032] 0.010	0.545 [0.121–0.801] 0.058
ULF SIENAX (T1)	− 0.377 [− 0.649 – − 0.020] 0.067	0.528 [0.097–0.792] 0.058
ULF WMH-SS	** − 0.568** **[− 0.771 – − 0.261]** **0.012**	**0.579** **[0.170–0.818]** **0.047**
Ventricular volume		
3 T SIENAX	0.295 [− 0.074–0.592] 0.143	− 0.423 [− 0.736–0.039] 0.099
3 T FreeSurfer	0.230 [− 0.142–0.545] 0.222	− 0.469 [− 0.761 – − 0.019] 0.083
ULF SIENAX	0.380 [0.023–0.651] 0.067	− 0.324 [− 0.678–0.153] 0.194
ULF WMH-SS	0.361 [0.001–0.638] 0.078	− 0.432 [− 0.741–0.028] 0.095

The analysis was performed using partial correlations (non-parametric for EDSS, parametric for SDMT), adjusted for age, sex, and the version of the form used for SDMT. ATOPS was assessed only in six healthy controls and 16 individuals with multiple sclerosis and was not utilized for partial correlation analysis

For tissue volume comparisons, either head size for SIENAX measures or total intracranial volume for WMH-SynthSeg measures was included as an additional covariate. In bold are shown significant *p* values < 0.05 that survived the false discovery rate (FDR) correction with the Benjamini–Hochberg procedure. FDR correction was applied across all imaging measures for a given clinical outcome

*CI* confidence interval, *EDSS* Expanded Disability Status Scale, *SDMT* Symbol Digit Modalities Test, *ULF WMH-SS* ultra-low-field WMH-SynthSe

### Relationship between clinical outcomes and MRI measures in Cohort 2 (ULF in severe vs. less severe MS)

Neither T2-LV nor T1-LV measures showed significant associations with any of the assessed disability measures. All SIENAX-derived measurements were significantly related to clinical outcomes, except for WM volume with respect to SNRS, Combi-WISE, and ATOPS. Unlike with SIENAX, WMH-SynthSeg WM volume significantly correlated with all clinical outcomes. WMH-SynthSeg-derived cortical GM volume correlated only with SDMT (Table 5) (*r* = 0.415).

**Table 5:** Correlations between clinical outcomes and MRI measures in people with multiple sclerosis in Cohort 2

MRI-based measures	EDSS Correlation [95% CI] *p* value	SNRS Correlation [95% CI] *p* value	Combi-WISE Correlation [95% CI] *p* value	SDMT Correlation [95% CI] *p* value	ATOPS Correlation [95% CI] *p* value
T2-lesion volume					
Semi-automated	0.142 [− 0.195–0.450] 0.482	− 0.170 [− 0.472–0.168] 0.349	0.145 [− 0.197–0.457] 0.438	− 0.256 [− 0.555–0.102] 0.170	0.208 [− 0.134–0.506] 0.249
ULF WMH-SS (FL)	0.192 [− 0.145–0.490] 0.340	− 0.274 [− 0.553–0.060] 0.153	0.215 [− 0.128–0.511] 0.280	− 0.265 [0.092–0.143] s0.169	0.282 [− 0.057–0.562] 0.133
T1-lesion volume					
Semi-automated	0.080 [− 0.256–0.398] 0.694	− 0.077 [− 0.396–0.258] 0.655	0.045 [− 0.293–0.372] 0.799	− 0.181 [0.498–0.179] 0.323	0.126 [− 0.216–0.441] 0.470
ULF WMH-SS (T1)	0.196 [− 0.141–0.493] 0.340	− 0.174 [− 0.475–0.164] 0.349	0.150 [− 0.193–0.460] 0.438	− 0.273 [− 0.568–0.083] 0.169	0.281 [− 0.058–0.561] 0.103
Whole brain volume					
ULF SIENAX	** − 0.618** **[− 0.789 – − 0.358]** **0.001**	**0.451** **[0.138–0.682]** **0.027**	** − 0.542** **[0.744 – − 0.249]** **0.006**	**0.518** **[0.200–0.737]** **0.006**	** − 0.502** **[− 0.718 – − 0.198]** **0.013**
ULF WMH-SS (T1)	** − 0.472** **[− 0.696 – − 0.164]** **0.018**	**0.481** **[0.176–0.702]** **0.027**	** − 0.497** **[− 0.715 – − 0.191]** **0.012**	**0.722** **[0.494–0.857]** ** < 0.001**	** − 0.530** **[− 0.736 – − 0.233]** **0.013**
Cortical gray matter volume					
ULF SIENAX	** − 0.598** **[− 0.776 – − 0.331]** **0.001**	**0.439** **[0.123–0.673]** **0.027**	** − 0.553** **[− 0.751 – − 0.264]** **0.007**	**0.530** **[0.216–0.744]** **0.006**	** − 0.481** **[− 0.705 – − 0.171]** **0.013**
ULF WMH-SS (T1)	− 0.067 [− 0.393–0.271] 0.694	0.284 [− 0.055–0.564] 0.153	− 0.247 [− 0.540–0.099] 0.229	**0.415** **[0.071–0.671]** **0.029**	− 0.229 [− 0.526–0.118] 0.228
Thalamic volume					
ULF WMH-SS (T1)	− 0.251 [− 0.539–0.090] 0.237	0.231 [− 0.111–0.524] 0.236	− 0.330 [− 0.601–0.009] 0.092	**0.626** **[0.349–0.802]** **0.001**	** − 0.408** **[− 0.656 – − 0.081]** **0.036**
White matter volume					
ULF SIENAX	** − 0.413** **[− 0.656 – − 0.093]** **0.044**	0.306 [− 0.031–0.580] 0.153	− 0.374 [− 0.632 – − 0.041] 0.054	**0.426** **[0.085–0.678]** **0.027**	− 0.329 [− 0.601–0.010] 0.093
ULF WMH-SS (T1)	− 0.348 [− 0.610 – − 0.016] 0.080	**0.402** **[0.079–0.648]** **0.044**	** − 0.413** **[− 0.659 – − 0.087]** **0.045**	**0.652** **[0.387–0.817]** ** < 0.001**	** − 0.492** **[− 0.711 – − 0.184]** **0.013**
Ventricular volume					
ULF SIENAX	0.390 [0.065–0.640] 0.053	− 0.292 [− 0.569–0.046] 0.153	0.388 [0.058–0.642] 0.050	** − 0.635** **[− 0.808 – − 0.362]** **0.001**	**0.438** **[0.117–0.676]** **0.025**
ULF WMH-SS (T1)	0.344 [0.012–0.608] 0.080	** − 0.443** **[− 0.676 –** − **0.129]** **0.027**	0.406 [0.078–0.654] 0.045	** − 0.427** **[− 0.679 – − 0.086]** **0.027**	**0.397** **[0.068–0.648]** **0.038**

*CI* confidence interval, *EDSS* Expanded Disability Status Scale, *SNRS* Scripts Neurological Rating Scale, *Combi-WISE* Combinatorial Weight-Adjusted Disability Score, *SDMT* Symbol Digit Modalities Test, *ATOPS* Auditory Test of Processing Speed, *ULF* ultra-low-field, *WMH-SS* WMH-SynthSeg, *T1* T1-weighted, *FL* T2-weighted fluid attenuated inversion recovery

The analysis was performed using partial correlations (non-parametric for EDSS, parametric for others), adjusted for age and sex. For tissue volume comparisons, either head size for SIENAX measures or intracranial volume for recon-all-clinical and WMH-SynthSeg measures was included as an additional covariate. All cells are displayed as correlation coefficients (*p* values). In bold are shown significant *p* values < 0.05 that survived False Discovery Rate (FDR) correction with the Benjamini–Hochberg procedure. FDR correction was applied across all imaging measures for a given clinical outcome

### Bland–Altman plots

**Fig. 3:**
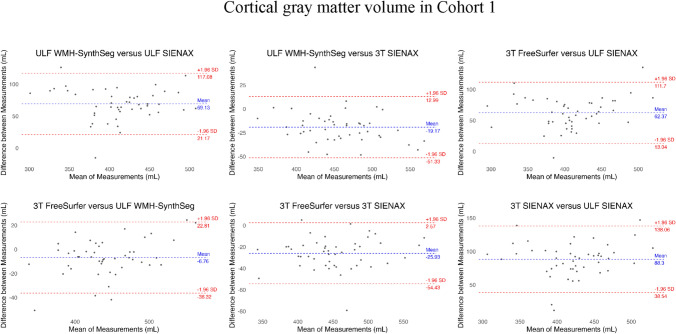
The Bland–Altman plots show comparisons between cortical gray matter volume obtained from ultra-low-field MRI and 3 T using different post-processing techniques. SIENAX was used for data from both field strengths while FreeSurfer was used only with 3 T and WMH-SynthSeg was used only with ultra-low-field MRI acquisitions. The blue line indicates the mean difference while the red lines indicate the limits of agreement, defined as the mean difference ± 1.96 standard deviations (SD)

**Fig. 4:**
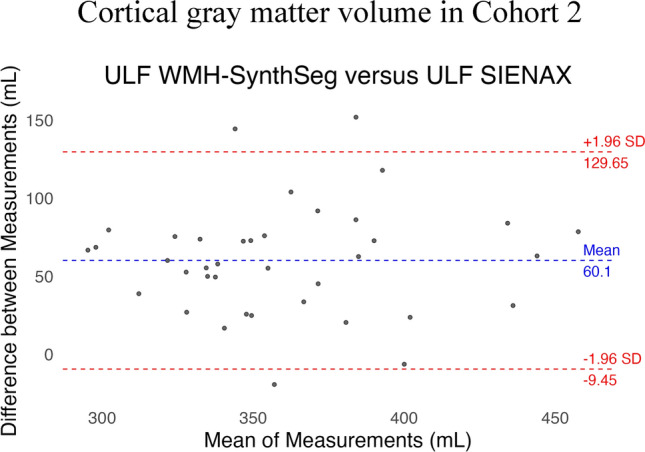
The Bland–Altman plot shows the comparison between cortical gray matter volume obtained from ultra-low-field MRI using SIENAX and WMH-SynthSeg. The blue line indicates the mean difference while the red lines indicate the limits of agreement, defined as the mean difference ± 1.96 standard deviations (SD)

Bland–Altman plots for cortical GM volume are shown for Cohorts 1 and 2 in Figs. 3 and 4. In Cohort 1, cortical GM volumes from the different post-processing methods and two different MRI systems showed generally small mean biases, with most measurements falling within the 95% limits of agreement. While variability differed slightly between comparisons, there was no strong evidence of proportional bias, although there was moderate inter-method variability at the individual level. In Cohort 2, the plot comparing ULF SIENAX and ULF WMH-SynthSeg showed a small systematic bias between the two methods, with most differences falling within the 95% limits of agreement. There was no clear proportional bias across the measurement range, although with variable levels of agreement at the individual level.

## Discussion

A recent international panel recognized that clinical trial designs, especially in severe MS, need to be more novel, use alternative approaches that would reduce sample size requirements, and prioritize development and application of intermediate outcomes and new technologies in under-researched MS populations [31]. Portable ULF MRI represents a near ideal solution for studying this patient population. We first compared results derived from 3 T acquisitions and ULF MRI. Next, we conducted a study where we brought portable ULF MRI into a specialized care facility for pwMS. The findings from our study support preliminary data [4, 32], showing lower brain volumes in those affected by severe MS compared to those with a milder form of the disease.

In validating ULF MRI, we found robust differences between HCs and pwMS. In terms of lesion assessment, we found the largest effect size for T2 LV using a fully automated method with ULF data. For T1 LV, we found similar effect sizes for both semi-automated and automated ULF measures. However, WMH-SynthSeg with T1w images resulted in a mean T1 LV of 3.0 mL in HCs, highlighting the risk of false positives. We also found that the ULF-derived tissue measures typically showed stronger associations with EDSS and SDMT compared to those obtained using 3 T MRI. For cortical GM volume, only ULF SIENAX showed a significant association with SDMT after multiple comparison correction. The reason for this discrepancy remains unknown and warrants additional investigation, especially since we found robust clinical associations with 3 T thalamic volumes, as expected [32, 33]. Regardless, findings from Cohort 1 support the use of ULF volumetry for research in pwMS.

When investigating Cohort 2, the largest effect sizes comparing pwMS with less versus more severe disease were noted for volumes obtained from the SIENAX pipeline, which yields a tissue segmentation based purely on image intensity along with registration-based masks to isolate the cortex and ventricles. Furthermore, cortical GM volume differences were only evidenced with SIENAX (partial η^2^** = **0.349, *p* = 0.003); the AI-based WMH-SynthSeg method showed very weak differences (partial η^2^** = **0.0.025, p = 0.495). On the other hand, the former did not show significant differences for WM volume while the latter did. Given that GM pathology is well known to be more strongly associated with increased disability and more advanced disease compared to that in the WM [34–37], we interpret our SIENAX-derived findings as having face validity. It is likely that the WMH-SynthSeg technique results in volumes being driven towards the mean, rendering it more difficult to detect real biological differences across two groups of pwMS already affected by substantial tissue atrophy. Although a study validated ULF MRI with WMH-SynthSeg in Alzheimer’s disease [38], it is conceivable that more subtle differences in cortical atrophy are lost when studying variability in different groups of pwMS. This issue was likely less of a problem in Cohort 1, where HCs were compared to pwMS.

We did not detect significant differences between lesion burden in severe versus less severe MS, as previously reported [4, 32], and between lesion burden and brain volumes in severe MS when comparing early versus late accrual of disability [4]. These findings may suggest that despite initial differences in demographic characteristics and clinical manifestations, early and late onset severe phenotypes ultimately converge on a common pathophysiological substrate characterized by GM-based neurodegeneration, as recently shown [4]. A likely cause of GM atrophy in early disability accrual severe MS is primary GM damage [34], whereas GM tissue loss in late severe MS may be the combination of primary [34], and secondary damage (WM lesions leading to secondary retrograde Wallerian degeneration) [39].

In the CASA-MS phase 1 study, it was found that dynamic disability measures (SNRS and Combi-WISE) were more robustly related to MRI outcomes in severe MS compared to the widely used EDSS [2]. In the current study, using the entire study population of severe and less severe pwMS in Cohort 2, we showed similar associations between increased disability and lower volumes of total GM, cortex (SIENAX only), thalamic (only feasible with WMH-SynthSeg), and WM (WMH-SynthSeg). We also previously characterized cognitive performance in severe and less severe MS using a newly developed and validated auditory cognitive test (ATOPS), thus eliminating the visual acuity, visual-motor, and memory confounds for which standard testing is criticized in the MS literature [3]. In line with previous results [3], a significantly greater percentage of severe MS completed ATOPS than the current gold standard of SDMT (95.8% vs 79.2%). All in all, these findings suggest that ULF MRI brain volumetry is useful for investigating clinico-radiological associations in MS.

Our study is not without its limitations. First, our ULF imaging protocol did not use isotropic voxels, which may have had an influence on the AI-derived cortical segmentations; it has been shown that hippocampal volumes obtained from ≤ 3 mm isotropic ULF MRI using these methods are more accurate than images obtained with anisotropic voxels [38]. The thick slices used in the ULF protocol (i.e., 5 mm) likely affected the volumetric estimates due to partial voluming effects; this could explain why we did not see significant group differences between HCs and pwMS in Cohort 1 for ventricular volume. Moreover, differences in the effects of slice thickness on segmentation quality and reliability across different algorithms may have had an impact on the degree of inter-method variability, as shown in the Bland–Altman plots. Although that analysis showed limited bias, at least for cortical volume, the differences appear to be clinically relevant, as evidenced by differential associations with clinical outcomes between different segmentation techniques. Second, our analyses were limited to cross-sectional data, preventing us from drawing firm conclusions on the utility of ULF in tracking atrophy. Future studies should optimize longitudinal processing pipelines designed specifically for ULF acquisitions. We also acknowledge that the sample sizes of the individuals early and late severe MS groups are quite modest, resulting in comparisons that are likely underpowered and thus risking type II error. This is especially notable for conventionally derived WM volume, where a large effect size was seen despite not being significant after correction for multiple comparisons. Finally, we did not consider the role of DMT status or history in our statistical analysis. More than 80% of the less severe MS group were on either moderate (50%) or high efficacy (18.8%) therapy compared to no therapy (87.5%) or moderate efficacy (12.5%) in the severe MS group. Matching the groups with respect to treatment history was not possible, rendering DMT status inextricably linked to severity status in our study participants. With an average disease duration of 30 years, on the one hand, many pwMS with severe disease did not have access to modern high efficacy treatments, which likely could have altered their disease course leading to more favorable clinical and imaging outcomes. On the other, patients in the less severe group with longstanding disease, by definition, had a milder form of the disease and likely benefited from the more modestly efficacious treatments historically available. Overall, studies with larger sample sizes are warranted to confirm our findings and allow for closer matching between groups, including in terms of DMT history.

In conclusion, our study validates the use of ULF MRI in assessing pwMS. Moreover, the CASA-MS phase 2 study demonstrates the feasibility of using ULF portable MRI in studying severe MS. We found associations with ULF-derived tissue volumetry and clinical endpoints across a broad spectrum of disease severity, suggesting their potential for assessing neurodegeneration and disability progression in those individuals living with severe MS.

## Supplementary Information

Below is the link to the electronic supplementary material.

Supplementary file1 (DOCX 17 KB)
