# Supplementary material for: Ultra-low-field MRI for bedside imaging of severe multiple sclerosis
Source: J Neurol. 2026 Jun 3;273(6):364. doi: 10.1007/s00415-026-13907-w (PMC13234000; doi:10.1007/s00415-026-13907-w)
Supplement: Supplementary file 1 — Supplementary file1 (DOCX 17 KB) [file 415_2026_13907_MOESM1_ESM.docx]

**Supplement Table.** Correlation coefficients between different segmentations techniques and field strengths

|  | 3T SIENAX | 3T FreeSurfer | ULF SIENAX | ULF WMH-SS |
| --- | --- | --- | --- | --- |
| Whole brain volume | | | | |
| 3T SIENAX | - | 0.958  (< 0.001) | 0.909 (< 0.001) | 0.939 (< 0.001) |
| 3T FreeSurfer | 0.958  (< 0.001) | - | 0.867 (< 0.001) | 0.942 (< 0.001) |
| ULF SIENAX | 0.909 (< 0.001) | 0.867 (< 0.001) | - | 0.914 (< 0.001) |
| ULF WMH-SS | 0.939 (< 0.001) | 0.942 (< 0.001) | 0.914 (< 0.001) | - |
| White matter volume | | | | |
| 3T SIENAX | - | 0.936 (< 0.001) | 0.818 (< 0.001) | 0.844 (< 0.001) |
| 3T FreeSurfer | 0.936 (< 0.001) | - | 0.762 (< 0.001) | 0.883 (< 0.001) |
| ULF SIENAX | 0.818 (< 0.001) | 0.762 (< 0.001) | - | 0.837 (< 0.001) |
| ULF WMH-SS | 0.844 (< 0.001) | 0.883 (< 0.001) | 0.837 (< 0.001) | - |
| Cortical gray matter volume | | | | |
| 3T SIENAX | - | 0.961 (< 0.001) | 0.872 (< 0.001) | 0.903 (< 0.001) |
| 3T FreeSurfer | 0.961 (< 0.001) | - | 0.880 (< 0.001) | 0.906 (< 0.001) |
| ULF SIENAX | 0.872 (< 0.001) | 0.880 (< 0.001) | - | 0.876 (< 0.001) |
| ULF WMH-SS | 0.903 (< 0.001) | 0.906 (< 0.001) | 0.876 (< 0.001) | - |
| Ventricular volume | | | | |
| 3T SIENAX | - | 0.983 (< 0.001) | 0.987 (< 0.001) | 0.982 (< 0.001) |
| 3T FreeSurfer | 0.983 (< 0.001) | - | 0.962 (< 0.001) | 0.988 (< 0.001) |
| ULF SIENAX | 0.987 (< 0.001) | 0.962 (< 0.001) | - | 0.974 (< 0.001) |
| ULF WMH-SS | 0.982 (< 0.001) | 0.988 (< 0.001) | 0.974 (< 0.001) | - |

**Legend:** ULF – ultra-low-field; WMH-SS – WMH-SynthSeg

Cells are shown as Pearson correlations (p-value).
